# Characterization of a New Rhamnolipid Biosurfactant Complex from *Pseudomonas* Isolate DYNA270

**DOI:** 10.3390/biom9120885

**Published:** 2019-12-17

**Authors:** Gina S. Shreve, Ronald Makula

**Affiliations:** 1 Department of Chemical Engineering and Material Sciences, Wayne State University, Detroit, MI 48202, USA; 2 Department of Biochemistry, University of Georgia, Athens, Ga 30302, USA; shreve1@earthlink.net

**Keywords:** rhamnolipid production, glycolipid, surface activity, growth physiology, interfacial activity, rhamnolipid characterization

## Abstract

The chemical and physical properties of extracellular rhamnolipid synthesized by a nonfluorescent Pseudomonas species soil isolate, identified as DYNA270, is described, along with characteristics of rhamnolipid production under varying growth conditions and substrates. The biosurfactant is determined to be an anionic, extracellular glycolipid consisting of two major components, the rhamnopyranoside β-1-3-hydroxydecanoyl-3-hydroxydecanoic acid (GU-6) and rhamnopyranosyl β→β2-rhamnopyranoside-β1-3-hydroxydecanoyl-3-hydroxydecanoic acid (GL-2), of molecular weight 504 and 649 daltons, respectively. These glycolipids are produced in a stoichiometric ratio of 1:3, respectively. The purified rhamnolipid mixture exhibits a critical micelle concentration of 20 mg/L, minimum surface (air/water interface) tension of 22 mN/m, and minimum interfacial tension values of 0.005 mN/m (against hexane). The pH optimum, critical micelle concentration, and effective alkane carbon number were established for Pseudomonas species DYNA270 and compared to those of rhamnolipid produced by *Pseudomonas aeruginosa* PG201. Significant differences are documented in the physical properties of extracellular rhamnolipids derived from these two microorganisms. The surface properties of this rhamnolipid are unique in that ultra-low surface and interfacial tension values are present in both purified rhamnolipid and culture broth containing the rhamnolipid complex (GU6 and GL2). We are not aware of prior studies reporting surface activity values this low for rhamnolipids. An exception is noted for an extracellular trehalose glycolipid produced by Rhodococcus species H13-A, which measured 0.00005 mN/m in the presence of the co-agent pentanol (Singer et al. 1990). Similar CMC values of 20 mg/L have been reported for rhamnolipids, a few being recorded as 5–10 mg/L for Pseudomonas species DSM2874 (Lang et al. 1984).

## 1. Introduction

Biosurfactants are surface active products derived from biological sources, which, like synthetic surfactants, possess the ability to dramatically lower interfacial tensions. Surface-active molecules of biological origin occur either as cell-bound structural entities or accumulate as extracellular bioproducts. Early reports of extracellular biosurfactants include sophorolipids from *Torulopsis bombicola* [1,2] rhamnolipid from *Pseudomonas aeruginosa* [2,3,4,5] surfactin from *Bacillus subtilis* [6] trehalose mycolates [7] and trehalose tetraesters from *Rhodococcus erythropolis* [7], and trehalose mycolates from Rhodococcus species H13A [8]. Other biological structures with surface activity include phospholipids, sugar lipids, and certain antibiotics [7,9,10], Rhamnolipid biosurfactant structures and origins have been recently reviewed [11], and interest in their production and properties remains strong.

There is significant interest in biosurfactants due to their ecological acceptability, variety of potential environmental applications [12,13], and ability to be produced through growth on renewable feedstocks, including low-value feedstocks, sometimes including waste streams [14]. This report describes the physical and chemical properties of an extracellular anionic glycolipid with unique surface properties synthesized by a newly isolated Pseudomonas species, DYNA270. As rhamnolipids are typically harvested from culture media where the biosurfactant-producing microorganisms may be grown in a process run under a variety of nutrient, pH, and substrate conditions, the rhamnolipid production from this soil isolate and the associated culture broth properties were closely examined during growth on a number of different carbon sources under varying pH and nutrient conditions.

## 2. Materials and Methods

### 2.1. Organism and Culture Conditions

An interfacial tension (IFT)-based screening procedure yielded a Gram-negative soil isolate that produced an extracellular surface-active product capable of lowering the interfacial tension to less than 0.05 mN/m when measured against crude oil. The soil isolate was identified as a nonfluorescent Pseudomonas species by using taxonomic identification protocols API 20B and API 20E, as previously described [15], and was designated as strain DYNA270. The basal salts minimal-growth medium (BSE) was (in gms/L): K_2_HPO_4_, 10; NaH_2_PO_4_, 5; (NH_4_)_2_SO_4_, 2; MgSO_4_.7H_2_O, 0.2; CaCl_2_.2H_2_O, 0.001; and FeSO_4_, 0.001, adjusted to pH 7.2. BSE medium was supplemented with the following sole sources of carbon and energy at the concentrations designated: decane through octadecane, 2% *v*/*v*; glucose, sucrose, and mannitol, 4% *w*/*v*; sodium palmitate, 0.075% *w*/*v*; sodium acetate or sodium succinate, 1.5% *w*/*v*; corn oil or canola oil, 1.5% *v*/*v*. These carbon-source concentrations were selected as typical for growth of Pseudomonas sp. on these various classes of carbon sources representing hydrocarbon, sugar, fatty acid, organic acids, or oils. Cell growth was determined by optical density at 590 nm in a Milton Roy Spectronic 20D. The ammonium ion concentration in the culture medium was determined by measuring the ammonia released from the medium, following adjustment to pH 11 with 10 M of NaOH, using an Orion ammonia-sensing gas electrode and a Fisher Accumet Model 500 selective ion analyzer.

### 2.2. Measurement of Surface and Interfacial Activity

Surface tension measurements were determined with a Fisher autotensiomat equipped with a denuoy ring. Interfacial tension measurements were performed on a University of Texas spinning drop tensiometer Model TX500K (Gaertner Scientific, Skolie, IL, USA), at 30 °C [16]. Interfacial tension measurements were made with either a Wyoming high-paraffin crude oil (API gravity 35) or pure *n*-alkanes.

### 2.3. Isolation of Glycolipid from Culture Broth

Crude lipid extracts were obtained by extraction of spent culture broth with 2 volumes of chloroform after removal of cells by centrifugation [8], and then they were fractionated by silicic acid column [17]. Neutral lipids were eluted with chloroform, surface-active glycolipids were eluted with acetone, and phospholipids were eluted with chloroform–methanol (2:1 *v*/*v*). Glycolipid was recovered in the acetone fraction, reduced to dryness, and reconstituted in 0.01 M of K_2_HPO_4_ buffer, pH 7.0. Glycolipids were quantified by the anthrone method, using rhamnose as the standard [18]. Glycolipid concentration is expressed in units of glycolipid dry weight. Glycolipid rhamnose content was correlated with glycolipid dry weight by weighing known amounts of glycolipid on a Cahn micro electrobalance with glycolipid of 1.0 mg dry weight being equivalent to 0.43 mg of glycolipid rhamnose. Reconstituted glycolipid exhibited interfacial tension values less than 0.05 mN/m, as determined by spinning drop tensiometry. No indication of either neutral lipid or phospholipid was noted in the spent culture broth, as determined by thin-layer chromatography.

### 2.4. Glycolipid Structural Analyses

Glycolipids were first separated by TLC on silica gel G thin-layer plates developed in chloroform/methanol/water-concentrated ammonium hydroxide (75:25:2:1, by volume) and were visualized by spraying a test strip with 75% sulfuric acid, followed by heating at 100 °C for 10 min. The two major glycolipids were purified by TLC on silica gel G thin-layer plates in the same solvent system and were eluted from the silica gel in chloroform/methanol/water (1:1:0.1, by volume) and reduced to dryness. Quantification of the 2 molecular species of rhamnolipid present in the culture broth was accomplished by spotting 120 µL of culture broth directly onto activated silica gel G thin-layer plates. The solvent system consisted of chloroform/methanol/water-concentrated ammonium hydroxide (75:25:2:1, by volume); a test strip was sprayed with 50% H_2_SO_4_ to locate the individual rhamnolipid species. The untreated silica gel areas containing the separated rhamnolipids were scrapped into screw-cap tubes and extracted 3 times with chloroform–methanol (1:2, by volume). All silica gel washings were pooled and reduced to dryness, and then the residue was reconstituted in a known volume of 0.01 M of phosphate buffer, pH 5.2.

#### 2.4.1. Carbohydrate Analysis

Linkage analyses of GL2 and GU6 glycolipid were performed by using the NaOH–methanol method [19]. The methylated samples were hydrolyzed in 2 M of trifluoroacetic acid at 121 °C for 2 h, and the hydrolyzed product was reduced with sodium borodeuteride at room temperature. The product was acetylated by using acetic anhydride at 120 °C for 3 h. The derivatized samples were analyzed by gas–liquid chromatography/mass spectrometry (GLC/MS) (Agilent Technologies, Santa Clara, CA, USA), using a Sp2330 Supelco column (Supelco Inc., Bellefonte, PA, USA). Internal standard (myoinositol) was added to each sample prior to the reduction step.

GL2 and GU6 were permethylated by the NaOH–methanol method, and the products were analyzed by MALDI-MS. MALDI-TOF-MS was performed with a Hewlett Packard 2025A mass spectrometer (Agilent Technologies, Santa Clara, CA, USA) operated in the positive-ion mode at 30 kV and a pressure of 6 × 10^−7^ torr. The mass spectrometer was calibrated with a mixture of glucose oligomers (degree of polymerization between 3 and 20). Aqueous solutions of samples were diluted 1:3 with aqueous 50% acetonitrile containing 100 mM of 2,5-dihydroxybenzoic acid. A sample (0.5 µL) was applied to the probe tip and desorbed with a nitrogen laser (337 nm), with a pulse width of 3 ns, and delivering approximately 16 ìJ of energy/laser pulse. The fatty acids are lost during the methylation procedure.

#### 2.4.2. Fatty Acid Analyses

Glycolipid fatty acids were analyzed by gas–liquid chromatography/mass spectrometry (GLC/MS) of fatty acid methyl esters (FAME) prepared by acid methanolysis of the glycolipid [17]. The FAME were analyzed by capillary GLC, using a Shimadzu GC-9A gas–liquid chromatograph (Shimadzu Scientific Instruments Inc, Columbia, MD, USA) equipped with a flame ionization detector. The FAME were separated on a 50 m × 0.2 mm fused silica capillary column coated with a cross-linked methylsilicone stationary phase (Hewlett Packard, Palo Alta, CA, Canada) with a temperature gradient of 80 to 300 °C. Peak identifications were based on the comparison of retention with standards. Peak areas were quantified by using methylnonadecanoate as the internal standard prior to sample analysis. GLC data were acquired and manipulated by a Nelson chromatography data system (Nelson Analytical Inc., Paramus, NJ, USA).

GLC/MS analyses were performed on a Hewlett Packard 5995A gas chromatograph–mass spectrometer equipped with a RTE 6/VM data system, using the same GLC conditions as described above. The temperatures of the transfer line, the source, and the analyzer were 300, 230, and 280 °C, respectively. The electron impact energy was 70 eV. Mass spectrometric identification of the FAME was based on a comparison of spectra with authentic standards and with spectra reported in the literature [20].

## 3. Results and Discussion

### 3.1. Extracellular Glycolipid Synthesis

Significant levels of extracellular glycolipid were produced during growth on alkanes, glucose, mannitol, corn oil, and canola oil, whereas reduced levels of extracellular glycolipid occurred following growth on organic acids, sucrose, ethanol, and palmitate (Table S1). Glucose-grown cultures exhibited extreme foaming in shake flasks and fermenters, resulting in culture and product loss from culture vessels. It was noted that mannitol-grown cells produced significantly less foaming, and extracellular yields of glycolipid were higher. Accordingly, the following studies were done with extracellular glycolipid derived from mannitol-grown cultures.

**Figure 1:**
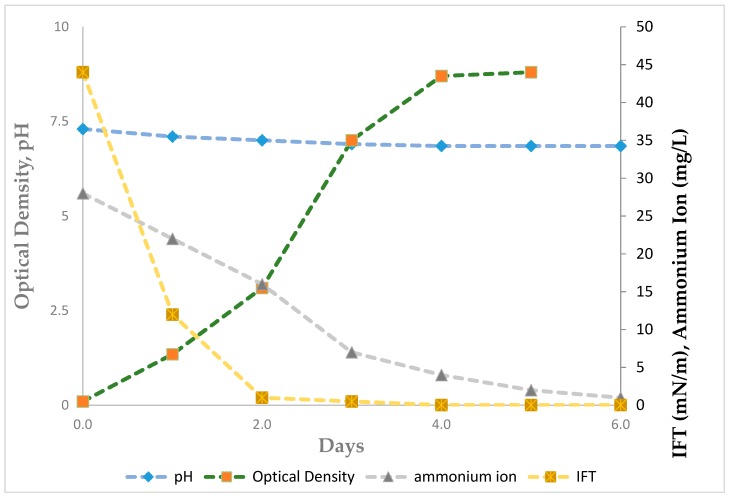
Interfacial tension, pH, and ammonium ion concentration during growth of *Pseudomonas* sp. DYNA270.

Figure 1 shows the relationship between cell growth on 4% mannitol and the lowering of IFT. Glycolipid biosurfactant (GL-2 and GU-6) concentrations increased approximately 30-fold over the culture period, accumulating 5.35 g/L in six days, for a specific production of 1.27 g glycolipid per gram dry cell weight (Table S1). No evidence was noted, by microscopic examination, of either cell lysis or cell aggregation. The increase in extracellular glycolipid was inversely correlated to the surface activity of the spent culture broth, with the interfacial tension decreasing from 47 to 0.002 mN/m against the high-paraffin crude oil in six days (Figure 1). Extracellular glycolipid production coincided with the depletion of ammonium ion concentration in the growth medium, with ammonium ion decreasing from 30 to 1.1 mM in six days (Figure 1). Initial ammonium ion concentrations of 60 and 90 mM resulted in increased cell densities of approximately 20% with a corresponding 70–80% decrease in extracellular glycolipid production.

Rhamnolipid production by Dyna270 was also examined on alternate nitrogen sources, and it was determined that ammonium sulfate allowed for constitutive synthesis of rhamnolipid throughout the culture period (Table S2).

### 3.2. Comparison of the Rhamnolipid Complex Surface Properties

A comparison of surface and interfacial tension values of purified rhamnolipid and cell-free culture broths containing GL2 and GU6 rhamnolipid from DYNA270 grown under varying conditions of pH, dilution, divalent cation, temperature, and 5% NaCl is shown in Table 1 and Table 2. Temperature-stability studies demonstrated that culture broths containing rhamnolipids were stable with respect to minimal surface activity values when autoclaved for 10 min at pH values of 6.5 and 7.0. When the pH values were either higher or lower, the surface activity was reduced in excess of 70%. Incubation of rhamnolipid-containing culture broths at 80 °C for 8 h resulted in no loss of ultralow surface-activity values. However, culture broths have been maintained at room temperature in excess of 2 years without any loss of surface activity or contamination by microorganisms (Table 1). An obligatory dilution of the culture broth is required in order to bring the IFTs assessed within the lower range of the tensiometer instrument used due to the ultralow surface-activity values (Table 1). This dilution factor is totally dependent on the concentration of the rhamnolipids present in culture broths to obtain these low interfacial tension values. Neither the divalent cations magnesium, iron, or calcium nor 5% NaCl affected the surface activity of the rhamnolipids.

### 3.3. Isolation and Identification of Extracellular Glycolipid

Thin-layer chromatography of the column-purified glycolipid demonstrated the presence of two major glycolipid species, designated GL2 and GU6, with R_f_ values of 0.20 and 0.62, respectively. The two glycolipid species were purified by preparative thin-layer chromatography for structure analyses. Column-purified glycolipid was hydrolyzed, and the carbohydrate component was analyzed by TLC. These results demonstrated rhamnose as the singular carbohydrate and, accordingly, the extracellular glycolipid was identified as a rhamnolipid. TLC of the purified rhamnolipid revealed two separate and distinct molecular species. The upper rhamnolipid (R_f_ = 0.62, GU6) measured 1.27 gms/L and lower rhamnolipid (R_f_ = 0.2, GL2) measured 3.74 gms/L for a total of 5.01 gms/L of extracellular rhamnolipid. A total recovery of 93.6% rhamnolipid resulted from the direct quantification of culture broth rhamnolipid (5.32 gms/L).

### 3.4. Chemical Properties of GL2 and GU6

Thin-layer chromatography of the deacylated glycolipid established rhamnose as the singular carbohydrate. Permethylation, followed by GLC/MS, showed GL2 to be rhamnopyranosyl β→β2-rhamnopyranoside, with a molecular mass of 309 daltons. GU6 was characterized as a β-rhamnopyranoside, with a molecular mass of 164 daltons. Gas–liquid chromatography of the fatty acid(s) recovered from GU6 and GL2 established identities as 3-hydroxydecanoic acid in both molecular species. Trace amounts of 3-hydroxydodecanoic acid were present in the purified samples. Molecular weights of GU6 and GL2 were established as 504 and 649 daltons, respectively.

### 3.5. Physical Properties of Rhamnolipid

Physical properties of the purified rhamnolipid derived from the culture broths of mannitol-grown cells were determined without separation of GL2 and GU6, unless otherwise indicated. Interfacial tension (IFT) measurements of GU6 and GL2 individually measured 0.499 and 0.81 mN/m, respectively, against high-paraffin crude oil (API35). However, when reconstituted together in 0.01 M of phosphate buffer, pH 5.2, minimum IFT values were dependent on the concentration of GU6 when GL2 was maintained at a constant concentration (Table 2). A concentration gradient of purified GU6 ranging from 20.57 to 242.84 μM, with GL2 at a constant concentration of 100 μM, yielded a minimal IFT of 0.006 mN/m at a GL2/GU6 ratio of 1:3, indicating the importance of GU6 to ultralow surface-activity values. The direct estimation of the culture broth concentration of rhamnolipid complex GU6 and GL2 was determined by spotting 120 μL of cell-free culture broth on a silica gel G TLC plate and resolving in chloroform/methanol/water-concentrated ammonium hydroxide (75:25:2:1, by volume). The individual rhamnolipids were eluted with chloroform–methanol (1:2, by volume), reduced to dryness, and then quantified. GU6 and GL2 were determined at representative concentrations of 3.08 and 5.95 mM, respectively, in the culture broth of mannitol-grown cells. The structures of GU6 and GL2 are identical to those previously described as rhamnolipid R1 and R2, respectively [21]. These rhamnolipids are also commonly referred to as mono- and di-rhamnose, with Β-hydroxydecanoyl-Β-hydroxydecanoate (Rha-C_10_-C_10_ and Rha-Rha-C_10_-C_10_), respectively. The rhamnolipid complex produced by the PG201 consisted of approximately two-fold more of the mono- than di-rhamnose C_10_-C_10_ glycoform. In contrast, DYNA270 produces a ratio of approximately 3:1 of di-rhamnose C_10_-C_10_ to the mono-rhamnose C_10_-C_10_ glycolipid congener. In this reported work, the stoichiometric ratio of these two glycolipid congeners is clearly demonstrated to dramatically influence resulting interfacial tensions (Table 2). This fact may, in part, explain the lower interfacial tensions and cmc generally obtained with DYNA270 as interfacial packing of surfactant head group molecules has been demonstrated to play a significant role in affecting final interfacial tension values achieved [22], as well as hydrocarbon substrate biodegradation preference for the organism [23], as a major ecological role for biosurfactant production in microbes is to facilitate degradation of insoluble substrate [24].

**Figure 2:**
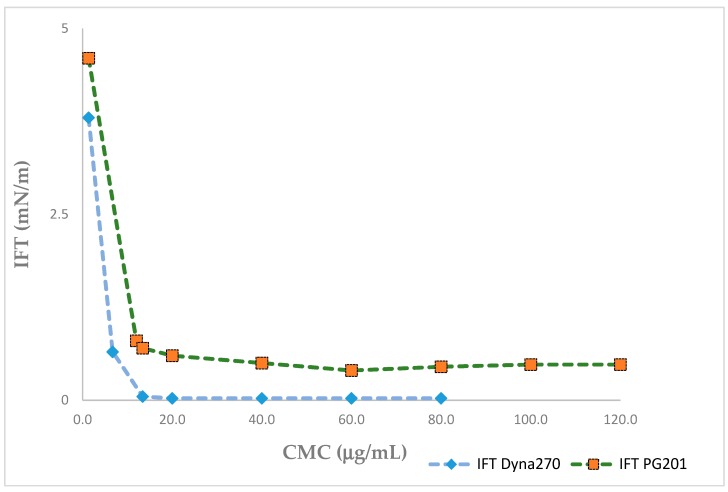
Critical micelle concentration (CMC) of PG201 rhamnolipid vs. DYNA270 rhamnolipid.

**Figure 3:**
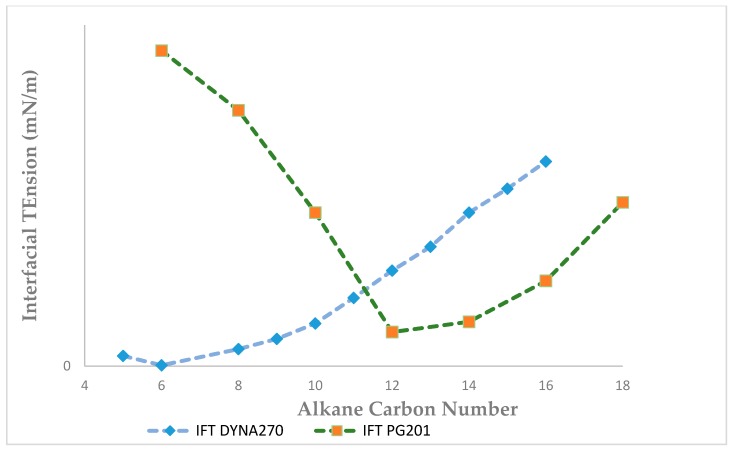
DYNA270 and PG201 effective alkane carbon number plot.

**Figure 4:**
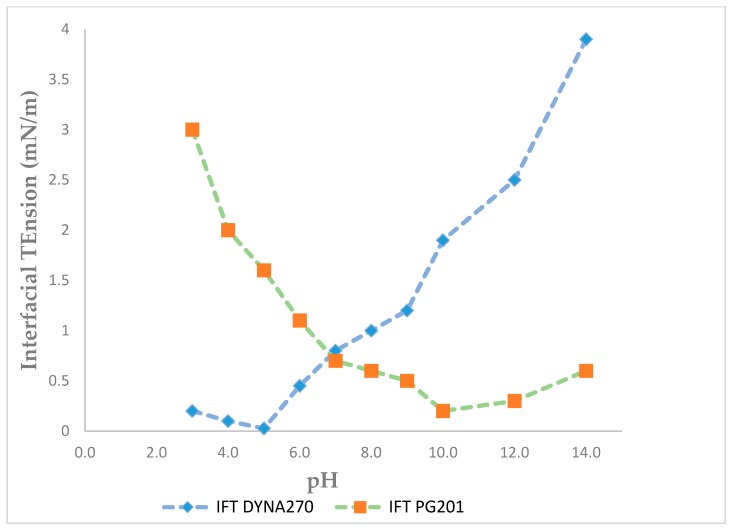
DYNA270 and PG201 interfacial tension as a function of pH.

The critical micelle concentration (CMC) of purified rhamnolipid derived from DYNA270 was 20 μg/mL, with a minimum IFT value of 0.005 mN/m at pH 5.2, as contrasted to PG201, with a minimum IFT value of 0.24 mN/m at 26 μg/mL (Figure 2). The IFT of the purified rhamnolipids were measured against a homologous series of *n*-alkanes ranging from pentane through hexadecane. A minimum IFT value of 0.006 mN/m was determined for hexane, with hexadecane measuring 0.6 mN/m for DYNA270, as contrasted to a minimum IFT of 0.085 mN/m for PG201 against dodecane (Figure 3). The DYNA270 optimum pH value was pH 5.2, yielding a minimum IFT value of 0.005 mN/m, as contrasted to PG201, which showed a minimum IFT value of 0.172 mN/m at pH 10.0 (Figure 4). The same physical performance properties were characteristic of the culture broths of DYNA270 and PG201, except that DYNA270 culture broths required dilution factors of 1:200, while PG201 culture broths could be diluted only 1:25, to maintain IFTs within the instrument ranges. 

## 4. Conclusions

We have described the general physiology of extracellular rhamnolipid synthesis by the soil isolate Pseudomonas species DYNA270. Two molecular species of extracellular rhamnolipid accumulate in the culture broth in unequal proportions, following growth on 4% mannitol and limiting ammonium ion concentrations. The benefit of growth on mannitol lies in less carbon dioxide evolution, less foaming, and increased yields of extracellular rhamnolipid throughout the fermentation time course. Quantification of these two molecular forms of rhamnolipid revealed a 1:3 ratio of mono-rhamnose C_10_-C_10_ to di-rhamnose C_10_-C_10_. 

We also described the physical and chemical properties of an extracellular biosurfactant synthesized by mannitol-grown *Pseudomonas* species DYNA270. The extracellular biosurfactant was identified as a rhamnolipid complex consisting of two rhamnolipid molecular species. Di-rhamnose with C_10_-C_10_ fatty acid (GL-2) was identified as rhamnopyranosyl β→β2-rhamnopyranoside-β2′-3-hydroxydecanoyl-3-hydroxydecanoic acid and the mono-rhamnose with C_10_-C_10_ fatty acid (GU6) was characterized as rhamnopyranoside β1-3-hydroxydecanoyl-3-hydroxydecanoic acid, structures previously reported in the literature [25]. While the surface properties of a number of rhamnolipids have been studied in some detail, our data is similar for some properties reported and significantly different from reported results of properties of other rhamnolipid mixtures (Table 3). The CMC values reported herein are consistent with those reported for Pseudomonas species DSM2874 [11], 25]. Also, the effect of divalent cations, as well as NaCl, is consistent with literature values. In contrast, Pseudomonas species DYNA270 exhibits an optimal pH value of 5.2, whereas Pseudomonas species DSM2874 was equally surface active at pH 3 and pH 9, as well as exhibiting low surface activity in distilled water. Pseudomonas species DYNA270 was inactive in distilled water, requiring ionic strength as 0.01 M of phosphate buffer, pH 5.2. Minimum interfacial tension values reported herein are in the low 10^−3^ mN range, under optimal conditions.

These studies further demonstrated that both molecular species of rhamnolipid are required for optimal surface activity. A summary of the physical properties of extracellular rhamnolipid complexes was shown in Table 2. Ultralow surface-activity values were also observed in magnesium chloride, iron sulfate, and calcium chloride at concentrations of up to 3 mM. Other performance parameters were stability at 120 °C at 18 psi (1 psi = 8.895 kPa), minimal surface activity of 0.002–0.005 mN/m at pH 5.2, and an equivalent alkane carbon number of hexane. This later parameter would indicate this rhamnolipid to have greater efficiency against high-paraffin-content crude oils. The concept of equivalent alkane carbon number (EACN) was developed to evaluate the effectiveness of different surfactant systems against crude oils containing complex mixtures of alkanes [26]. However, our studies have demonstrated the effectiveness of DYNA270 in displacing heavy oily sludges from polypropylene coupons (unpublished results). Further studies are necessary to resolve the significant differences that exist in the physical properties of rhamnolipid derived from different Pseudomonas species.

## Figures and Tables

**Table 1:** Surface and Interfacial Tension Properties of Rhamnolipid Derived from *Pseudomonas* species DYNA270.

Surfactant	Solution	MST ^1^ mN/m	CMC gm/L	MIFT ^2^ mN/m	CMC ^3^ gm/L
**Purified rhamnolipid [GL2 + GU6]**	distilled H_2_O	48	0.02	1.50	0.02
	0.01 M phosphate, pH 5.2	22	0.02	0.002	0.02
	0.01 M phosphate, pH 7.1	35	0.02	0.741	0.02
	0.01 M phosphate, pH 9.2	52	0.02	1.220	0.02
	0.01 M phosphate, pH 5.2, plus 5% NaCl	22	0.02	0.003	0.02
	0.01 M phosphate, pH 5.2, plus 3 mM Mg^2+^, Fe^2+^, Ca^3+^	22	0.02	0.004	0.02
**cell-free culture broth containing GL2&GU6**	distilled H_2_O	54	0.026	2.110	0.026
	autoclaved/10 min undilute	24	0.026	0.005	0.026
	undilute/90 °C, 8 h	25	0.026	0.004	0.026
	undilute, pH 5.2	34	0.026	1.310	0.026
	1:50 dilution ^4^ culture broth pH 5.2	28	0.026	0.091	0.026
	1:100 dilution culture broth, pH5.2	23	0.026	0.003	0.026
	1:150 dilute culture broth, pH5.2	22	0.026	00.003	0.026
	1:200 dilute culture broth, pH5.2	22	0.026	0.003	0.026
	1:200 dilute culture broth pH 5.2, plus 3 mM Mg^2+^, Fe^2+^, Ca^3+^	24	0.026	0.005	0.026
	1:200 dilution, culture broth, 5% NaCl	25	0.026	0.005	0.026

^1^ MST=minimum surface tension. ^2^ MIFT=minimum interfacial tension. ^3^ MgSO4.7H_2_O, FeSO4.7H_2_O, and CaCl_2_.2H_2_O were added individually at a 3 mM final concentration to 0.01 M phosphate buffer, pH 5.2. ^4^. All dilutions made in 0.01 phosphate buffer, pH 5.2.

**Table 2:** Titration of GU6 Rhamnolipid against GL2 Rhamnolipid.

Rhamnolipid Component	µMGL2	µMGU6	mN/m
GL2	---	14.8	0.499
GU6	100	----	0.810
GL2 + GU6	100	14.80	0.287
GL2 + GU6	100	20.57	0.171
GL2 + GU6	100	42.85	0.115
GL2 + GU6	100	84.88	0.078
GL2 + GU6	100	129.97	0.006
GL2 + GU6	100	71.42	0.025
GL2 + GU6	100	219.27	0.046
GL2 + GU6	100	242.84	0.095

**Table 3:** Summary of physical properties for three different rhamnolipid synthesizing microorganisms.

Property	*P*. species DYNA270	*P. aeruginosa* PG201	*P*. species DSM2874 ^a^
pH optimum	5.2	10.0	6.7–6.8
CMC	20 g/mL	26 g/mL	20 g/mL
EACN ^b^	hexane	dodecane	not reported
minimum IFT	0.005 mN/m	0.172 mN/m	<1 mN/m
dilution factor ^c^	1:200	1:25	not reported

^a^ data taken from Slydatk et. al. 1985. ^b^ EACN= equivalent alkane carbon number. ^c^ the dilution factor associated with each culture broth to maintain optimal surface activity values.

## Supplementary Materials

The supplementary materials are available online at https://www.mdpi.com/2218-273X/9/12/885/s1.
