# Supplementary material for: Characterization of a New Rhamnolipid Biosurfactant Complex from Pseudomonas Isolate DYNA270
Source: Biomolecules. 2019 Dec 17;9(12):885. doi: 10.3390/biom9120885 (PMC6995601; doi:10.3390/biom9120885)
Supplement: Supplementary file 1 [file biomolecules-09-00885-s001.pdf]

SUPPLEMENTARY INFORMATION:

Table SI. Extracellular Glycolipid Synthesis by DYNA270 on Various Carbon Sources

CARBON SOURCE	GLYCOLIPID (G/LITER)	GLYCOLIPID (G/G DRY CELL WT)
DECANE	1.51	0.359
DODECANE	1.54	0.366
TETRADECANE	1.69	0.401
HEXADECANE	1.79	0.425
OCTADECANE	1.08	0.257
GLUCOSE	2.28	0.542
MANNITOL	5.35	1.270
SUCROSE	0.18	0.042
ACETATE	0.16	0.038
SUCCINATE	0.08	0.019
ETHANOL	0.12	0.029
CORN OIL	3.05	0.724
CANOLA OIL	3.55	0.843
PALMITATE	0.17	0.040

Rhamnolipid Production by Dyna270 on Alternative Sources of Nitrogen.

A study was initiated of other nitrogen sources such as bound organic nitrogen, since rhamnolipid synthesis appears regulated by limiting nitrogen concentration. Sole sources of nitrogen analyzed were glycine and urea to determine whether increased levels of extracellular rhamnolipid allowed for the constitutive synthesis of rhamnolipid throughout the growth cycle (Table II).

These studies indicate that neither glycine nor urea stimulated the synthesis of extracellular rhamnolipid or allowed for the constitutive synthesis of rhamnolipid although cell yields were somewhat greater. In all cases, with either glycine or urea, the pH remained basic; whereas, with ammonium sulfate, the culture medium went acidic. Glycine at 0.02 M closely resembled ammonium sulfate augmented cultures, except for the pH values. Nitrogen sources such $NaNO_3$, NH_4NO_3, NH_4Cl, and glutamate also failed to increase rhamnolipid production over that obtained with $(NH_4)_2SO_4$ used in the published study medium.

Table S2. Effects of Bound Organic Nitrogen (0.02 M glycine, 0.03 M glycine, 0.66 M glycine, 0.05 M urea 0.01 M AS[1])

Hrs	A[2]	B[3]	C[4]	Hrs	A[2]	B[3]	C[4]	Hrs	A[2]	B[3]	C[4]
0	1.1	7.2	45	48	6.2	7.2	46	96	8.3	7.2	0.005
	1.9	7.2	46		8.3	7.2	46		9.1	7.2	45
	1.3	7.2	47		10.7	7.2	45		12.0	7.4	45
	1.1	7.2	46		8.6	7.4	45		11.0	7.5	45
	1.2	7.2	46		7.0	6.8	46		8.2	6.4	0.005
24	4.2	7.2	46	72	7.5	7.2	0.005				
	6.4	7.2	46		9.1	7.2	45				
	4.2	7.2	46		11.5	7.3	45				
	5.2	7.2	46		9.7	7.4	45				
	3.9	7.0	46		8.0	6.7	0.01				

1. AS=ammonium sulfate. 2. A=optical density at 590 mì. 3. B=pH. 4. C=IFT in mN/m.
